# Training to Improve Language Outcomes in Cochlear Implant Recipients

**DOI:** 10.3389/fpsyg.2013.00263

**Published:** 2013-05-14

**Authors:** Erin M. Ingvalson, Patrick C. M. Wong

**Affiliations:** ^1^Roxelyn and Richard Pepper Department of Communication Sciences and Disorders, Northwestern UniversityEvanston, IL, USA; ^2^Department of Otolaryngology – Head and Neck Surgery, Northwestern University Feinberg School of MedicineChicago, IL, USA; ^3^Department of Linguistics and Modern Languages, The Chinese University of Hong KongHong Kong SAR, China

**Keywords:** prelingual deafness, cochlear implant, language learning, auditory training, cognitive training

## Abstract

Cochlear implants (CI) have brought with them hearing ability for many prelingually deafened children. Advances in CI technology have brought not only hearing ability but speech perception to these same children. Concurrent with the development of speech perception has come spoken language development, and one goal now is that prelingually deafened CI recipient children will develop spoken language capabilities on par with those of normal hearing (NH) children. This goal has not been met purely on the basis of the technology, and many CI recipient children lag behind their NH peers with large variability in outcomes, requiring further behavioral intervention. It is likely that CI recipient children struggle to develop spoken language at NH-like levels because they have deficits in both auditory and cognitive skills that underlie the development of language. Fortunately, both the auditory and cognitive training literature indicate an improvement of auditory and cognitive functioning following training. It therefore stands to reason that if training improves the auditory and cognitive skills that support language learning, language development itself should also improve. In the present manuscript we will review the auditory and cognitive training and their potential impact on speech outcomes with an emphasis on the speech perception literature.

The advent of cochlear implants (CI) has brought with it the goal of spoken language performance on par with that of normal hearing (NH) listeners (Nicholas and Geers, 2007). This goal is not met purely on technology, requiring further behavioral intervention, and CI recipients are often found to lag behind their NH peers (Boothroyd et al., 1991; Dawson et al., 1995; Geers et al., 2009; Pisoni et al., 2010). Several factors have been identified to account for this lag, with age of implantation appearing to account for most of the variance (Geers, 2002, p. 200; Connor et al., 2006). Demonstrated in earlier reviews, pediatric CI recipients have deficits in those auditory and cognitive abilities that have been shown to be fundamental to language learning (Peterson et al., 2010), such as working-memory capacity and phonological awareness. In this review, we will revisit these auditory and cognitive deficits and how they relate to language learning, then demonstrate that training is effective for improving auditory and cognitive performance in both typically developing children and children with developmental delays. We conclude by discussing a preliminary effort to implement cognitive training in CI recipients.

## Auditory and Cognitive Deficits in CI Recipient Children

We begin by reviewing the auditory and cognitive deficits seen in CI recipient children (and we note that excellent, more extensive reviews of these deficits have been performed by (Nicholas and Geers, 2006; Peterson et al., 2010; Pisoni et al., 2010)). Because we are not the first to review these deficits, our aim here is not to comprehensively describe CI recipient children’s cognitive and auditory deficits. Instead, our aim is to orient the reader to these deficits relative to auditory and cognitive performance in NH children to support the claim that language deficits result because language depends on auditory and cognitive skills and to suggest that auditory and cognitive training could improve language performance. In the next section, we’ll review how these particular skills relate to language learning, suggesting that CI recipients’ language delays may be in part due to their auditory and cognitive deficits.

### CI recipient children show deficits in higher-level auditory skills

Because one aim of a CI is to allow recipient children to attain language performance on par with their NH peers (Nicholas and Geers, 2007), age of implantation has been made earlier to better mimic the age of hearing onset of NH children (Peterson et al., 2010). However, because the age of hearing onset is not identical to that of NH children (Forbes and Forbes, 1927) and because the type of hearing stimulation provided by the CI is not identical to the hearing NH children have (Krull et al., 2012), it is worth investigating whether the auditory skills of CI recipient children are delayed relative to their NH peers. The results indicate that CI recipient children are slower to recognize words and have poorer speech perception in noise (Grieco-Calub et al., 2009), are poorer with sound localization (Litovsky, 2011), and have greater variability in speech sound processing than their NH peers (James et al., 2008). Taking a closer look at the speech sound auditory skill subset, it seems that syllable awareness comes online first after implantation whereas rhyme and phoneme awareness are most delayed (James et al., 2005), with the ability to segment speech into its sound components still well below NH norms even after 4 years of CI use (Spencer and Tomblin, 2008). Even in those cases where CI recipients’ averages are found to fall near the range of NH children on auditory skills, the range of scores is larger than that for NH children (Dillon et al., 2012). Additionally, when CI recipient children’s receptive vocabulary is included in the model, the correlation between speech sound auditory skills and reading performance is much weakened, suggesting CI recipient children are relying on their lexicon for reading and sound processing strategies rather than an awareness of how speech sounds are segmented (James et al., 2009; Dillon et al., 2012).

It therefore appears that the delay in hearing onset does adversely affect CI recipient children, particularly in speech sound processing. However, these adverse effects may not be limited to speech perception. Early work investigating speech production in CI recipient children found that the rate of development was slower than that for speech perception, but that speech production increased steadily (Miyamoto et al., 1996; Blamey et al., 2001), though final attainment was not equivalent to the productions of NH children (Tobey et al., 2003). More recently, as CI technology has improved and implantation ages have moved younger, speech production growth rates have improved, particularly for early implantees (Connor et al., 2006; Ertmer and Goffman, 2011). Especially promising, when using amount of implant experience instead of chronological age as the referent, the majority of early CI recipients score within NH norms on measures of articulation (Flipsen, 2011). Though there continues to be variability in the rate of production acquisition and the order in which production sounds are acquired (Ertmer and Goffman, 2011), recent evidence suggests that CI recipient children lag behind their NH peers in production to a lesser extent than they do in perception. Because of this disparity, we will focus on speech perception for the remainder of this review. As we shall see in a later section, CI recipient children’s delays in speech sound processing can adversely affect language learning because those very skills that are impaired in CI recipient children have been shown to support language acquisition.

### CI recipient children show cognitive deficits

Because prelingually deafened children are implanted at early ages to better mimic the age of hearing onset of NH children, early implantation tends to result in better language outcomes than late implantation (Geers, 2002; Geers et al., 2007, 2008, 2009; Nicholas and Geers, 2007). However, even within this group of early implantees there is a great deal of variability in the outcomes (for a recent thorough review, see Peterson et al., 2010). Increasingly, researchers are looking to variability in CI recipients’ cognitive function as a possible means of accounting for variability in language outcomes. Pisoni and Cleary (2003) measured the digit spans of early-implanted CI recipients and age-matched controls. While all of the controls fell within the normal range for both backward and forward digit span, the CI recipients had spans that were noticeably reduced and the deficit was particularly pronounced for the forward digit span. Significant correlations were found between the rate of speech production – termed rehearsal speed – and digit span. Simply put, CI recipient children produced speech at a slower rate than NH children, and previously established relationships between speech production rate and inner speech rehearsal (Hitch et al., 1989) suggests that slower rehearsal speeds may lead to poorer maintenance of phonological information in working memory (Baddeley and Hitch, 1974), resulting in smaller digit spans for the CI recipient children.

Supplementing the digit span measure with another, non-word repetition, also shows that CI recipient children are not performing at the level of their NH peers. NH children were found to have a significant correlation between digit span and non-word repetition accuracy; these correlations were found to hold even when digit spans were reduced to the range found for the CI recipients. However, no relationship was found between digit span and non-word repetition accuracy for the CI recipient children, possibly due to a breakdown in the configuration of working-memory subcomponents (Watson et al., 2007). Whether stemming from reduced rehearsal speeds or poorer working-memory configuration – or, more likely, some combination thereof – the evidence clearly indicates that CI recipient children are not showing the same performance as their NH peers on measures of auditory working memory.

It is worth noting that while CI recipients perform more poorly than their NH peers on working-memory tasks that require a phonological component (Wass et al., 2008), many are within the normal range on other tests of cognitive ability (Dawson et al., 2002; Willstedt-Svensson et al., 2004). When the tests rely on visuospatial working-memory abilities, most CI recipient children perform within one standard deviation of published norms (Wass et al., 2008; Lyxell et al., 2009).

The disparity between CI children’s cognitive performance when the task is auditory compared to when the task is visuospatial has led to the suggestion that auditory input is essential to the development of auditory cognitive skills and that auditory deprivation early in life leads to cognitive deficits (Marshark, 1993; Conway et al., 2009). There is some support for this suggestion from the auditory aging literature. The observation that measures of auditory acuity were a strong predictor of age-related cognitive performance led to the suggestion that reduced perceptual input may be driving the reduced cognitive performance (Lindenberger and Baltes, 1994; Baltes and Lindenberger, 1997). A series of follow-ups have supported this finding (e.g., Choi et al., 2011; Rönnberg et al., 2011), including experiments that equate the perceptual experience of younger and older adults (e.g., Pichora-Fuller et al., 1995; Murphy et al., 2000; Schneider et al., 2000; Pichora-Fuller, 2003). These experiments have demonstrated that poor perception results in poorer cognitive performance, and that these effects are not limited to the auditory domain (e.g., Li et al., 2001; Cabeza et al., 2004). Though these data cannot definitively indicate that the absence of perception causes the cognitive deficits often seen in older adults or CI recipient children, they do suggest that the particular relationship between auditory deprivation and cognitive deficits in CI recipient children warrants further investigation to better understand the precise cause of the cognitive deficit.

## Auditory and Cognitive Skills are Linked to Language Learning

In the preceding sections we briefly reviewed the evidence indicating CI recipient children lag behind their NH peers on measures of auditory and working-memory functioning. Demonstrating a deficit is, however, insufficient to claim that these deficits are related to CI recipient children’s poorer performance on language measures relative to NH peers. We must also demonstrate that those skills in which the CI children have been shown to have a deficit are those skills on which language learning depends.

### Auditory skills are linked to language learning

In typically developing children, skills related to the ability to perceive and manipulate speech sounds have been found to be related to children’s language development. Infants’ ability to differentiate native-language vowels is predictive of word and phrase understanding (Tsao et al., 2004; Kuhl et al., 2005). Rhyme awareness, phoneme detection, and phoneme deletion have been found to be predictive of children’s vocabulary development (Bowey, 1996, 2001; Metsala, 1999). Children with language delays that manifest in delayed or reduced vocabulary or syntax development also show impaired speech perception skills (Leonard et al., 1992; Stark and Heinz, 1996). Suggesting that the observed auditory deficits are not specific to the speech perception mechanism, these same children also show impaired backward masking (Wright et al., 1997), though Rosen et al. (2009) demonstrated that the degree of masking impairment is not predictive of degree of language impairment. In a follow-up to these contrasting results, Nittrouer et al. (2011) suggest that children with language delays have deficits in creating robust categories from sensory information rather than a specific temporal deficit as demonstrated by backward masking, which allows for the deficit to be a general auditory impairment rather than speech-specific without a reliance on the temporal resolution of backward masking.

If we increase our definition of language development to include literacy development, it is now well established that the ability to process and manipulate the sounds of language is one of the best predictors of future literacy success for children (Foy and Mann, 2003; Hogan et al., 2005; Mann and Foy, 2007; Goodman et al., 2010). Tracking children through their first year of reading acquisition demonstrates that the ability to segment out individual sounds of speech sounds or understand rhyme in sounds prior to reading onset is the best predictor of later reading skills (Nithart et al., 2011). Screening children for difficulties segmenting the sounds of speech in kindergarten successfully predicts which children will have reading difficulties at the beginning of reading instruction (Bridges and Catts, 2011). Demonstrating that the connection between speech sound processing and language learning extends beyond typically developing children, rhyme awareness, and sound differentiation continue to be a significant predictor of reading ability even among children with low-IQ (Kuppen et al., 2011). Sound processing interventions have been shown to result in significant literacy improvements, suggesting these links are causal, not correlational, and that training could result in improved language learning (Hulme et al., 2012). This last point – that these links may be causal and that training can result in improved language learning – is especially encouraging because it means that interventions that improve CI recipient children’s ability to perceive speech may also improve their ability to learn language.

### Cognitive skills are linked to language learning

For typically developing children, working-memory capacity has been linked to vocabulary development (Adams et al., 1999; Willis and Gathercole, 2001; Geers et al., 2003) and syntax development (Adams and Gathercole, 1995). More specifically, 3-, 4-, and 5-year-old children’s non-word repetition abilities predicts their vocabulary size (Gathercole and Baddeley, 1989; Gathercole et al., 1992; Adams et al., 1999; van Daal et al., 2008; de Abreu et al., 2011), the length and syntactic complexity of utterances (Adams and Gathercole, 1995, 2000), and their expressive language abilities in a story-telling task (Adams and Gathercole, 1996). In terms of literacy, assessed prior to learning to read, phonological working memory is one of the best predictors of reading success (Seigneuric et al., 2000; Nevo and Breznitz, 2011). Indeed, those children who were identified as poor readers are also those that are found to have poor verbal working-memory spans relative to typical readers (Nation et al., 1999; Gathercole et al., 2006) though the two groups are equivalent on visuospatial spans (Nation et al., 1999). Working-memory capacity has also been shown to be important for the language development of children with language disorders, predicting their vocabulary and syntax development (Gathercole and Baddeley, 1990; Archibald and Joanisse, 2009; Anderson and Wagovich, 2010; Pierpont et al., 2011).

Looking specifically at CI recipient children, the non-word repetition task was the greatest predictor of word-learning and of both expressive and receptive grammar abilities (Willstedt-Svensson et al., 2004) and accounted for more variance than age of implantation, which is generally found to be the greatest predictor of language outcomes (Harrison et al., 2001; Geers, 2002; Connor et al., 2006; Bø Wie et al., 2007; Nicholas and Geers, 2007; Svirsky et al., 2007). Additionally, digit span correlates significantly with word recognition scores even after partialling out other variables that typically account for variability in language outcomes (Pisoni and Cleary, 2003). Given this connection between cognition and language learning for both typically developing and CI recipient children, it seems likely that CI recipient children’s language delay is due at least in part to their reduced cognitive performance.

## Auditory and Speech Perceptual Training

There are numerous laboratory studies demonstrating that auditory training can be effective for improving the auditory and speech perception abilities of NH adults. Because the aim of the current paper is to discuss the effectiveness of training for CI recipient children, and because the number of studies is too vast to be adequately discussed here, we leave it to the reader to look to other reviews to summarize the history of auditory training. We mention briefly the recent findings that a one-size-fits-all approach to auditory training may not be the optimal training approach because individual learners bring individual skills to the learning environment (Golestani et al., 2002, 2006; Wong and Perrachione, 2007; Wong et al., 2007, 2008; Song et al., 2008; Chandrasekaran et al., 2010; Perrachione et al., 2011). Thus, as we move into developing training paradigms to improve language outcomes for CI recipient children, the particular needs of the individual children may need to be considered, and personalized training may need to be developed.

Unfortunately for our purposes here, when looking to determine the effectiveness of auditory training in the CI population we are limited to a handful of studies done in CI recipient adults. In a series of single-subject designs, CI recipient adults received psychoacoustic pitch discrimination training, reduced-bandwidth telephone speech training, and speech-in-noise training (Fu and Galvin, 2008). All subjects significantly improved over multiple-baseline assessments; no control subjects were used. These single-subject studies addressed higher auditory difficulties typical of adult CI recipients (Fu and Galvin, 2006) and show that training can be effective to improving the auditory abilities of CI recipients.

With a larger sample size, Fu et al. (2005) trained CI listeners on those particular speech sounds that pretesting determined to be most difficult for each listener. After 16 weeks of adaptive training, all listeners showed significant improvements over baseline performance. Ingvalson et al. (2013) also used an adaptive training paradigm to improve the speech-in-noise perception of adult CI recipients, seeing significant improvements after only 4 days of training. Showing that the benefits of training can be extended to other language environments, Wu et al. (2007) found significant improvement in both speech sound and lexical tone identification over baseline following 10 weeks of adaptive training. None of these studies used a control group, though all three utilized multiple-baseline assessments prior to training. Though the small number of subjects and the lack of control groups make these studies with CI listeners less rigorous than those investigations of auditory training in NH adult listeners, they nonetheless provide preliminary evidence that auditory training can be effective for improving speech perception in CI recipients. However, there is the issue that these studies focused on the speech abilities of adult CI recipients, leaving the question of how effective auditory training will be in a pediatric population. Our best insight into the possible effectiveness of auditory training for children may come from auditory verbal therapy (AVT). Hearing impaired children, whether using hearing aids or CI, show rates of improvement on standardized measures of language that outpace what would be expected through normal development (Rhoades and Chisolm, 2001; Hogan et al., 2008, p. 2), though the multi-year duration of AVT, and the lack of a control group make it difficult to be certain if it is the therapy, and not development, that is driving the change (Eriks-Brophy, 2004; Rhoades, 2006). Recently, a more controlled study on AVT found significant improvements on receptive language, phonological awareness, articulation, and speech-in-noise perception (Fairgray et al., 2010). Again, the small sample size limits generalizability, but the significant improvement relative to baseline in a controlled setting suggests that AVT, or auditory training, could be effective for improving speech-and-language outcomes for CI recipients. More work using larger samples and control groups is needed to determine the effectiveness of auditory training for both CI recipient adults and children, but these preliminary data give us hope that those studies will result in better auditory and speech perception – and thereby better language abilities – in these populations.

## Cognitive Training

The preponderance of evidence supports the conclusion that there is a benefit to cognitive training for children, both those with a learning disability and those who are developing typically (Thorell et al., 2009; Klingberg, 2010; Morrison and Chein, 2010; Bergman Nutley et al., 2011; Diamond and Lee, 2011; Jaeggi et al., 2011). In a well-publicized effort to verify the link between cognitive training and improved cognitive function, Owen et al. (2010) collected pre- and post-training online measures of reasoning, verbal short-term memory, spatial working memory, and paired associates learning from 11,430 healthy adults. Following 6 weeks of online cognitive training (mean total training time was 4 h) that included the skills assessed, Owen et al. concluded that the benefits of training are minimal. However, this negative result can be attributed to a number of different factors, including the fact that many of the volunteers were likely cognitively healthy and the amount of training given was reduced relative to the studies in which training has been found effective, suggesting an underdosing of training for this population (Fisher et al., 2009; Vinogradov et al., 2012). Cognitive training typically focuses on a single component: cognitive training for working memory or cognitive training for attention and other aspects of executive functioning. We follow this trend and divide our review into those studies that train working memory and those studies that train attention and executive functioning. Above we noted emerging evidence in the auditory training domain that suggests training that takes the learner’s individual abilities into account may result in more optimal training outcomes. Similarly, the cognitive training data reveal that a program that adapts to the user’s performance as training progresses results in greater gains than training programs that are non-adaptive (Klingberg, 2010).

### Training working memory

The initial efforts to train working memory worked with children diagnosed with attention deficit hyperactivity disorder (ADHD) between 7 and 15 years old (Klingberg et al., 2002, 2005). Children trained on both visuospatial and verbal working-memory tasks, with an emphasis on visuospatial tasks, on an early version of Cogmed (Cogmed Systems, Stockholm, Sweden). Training was adaptive, with difficulty levels in subsequent sessions determined by performance in previous sessions; children’s starting levels were determined by their initial abilities. A control group completed a non-adaptive, easy version of the tasks. Both studies found a significant group × session interaction for trained and untrained tasks, suggesting a benefit of training. However, generalization was somewhat limited with children only showing a benefit on those tasks that tapped similar abilities to the training tasks (e.g., children did not show improvement on a reaction time task).

Further assessing the effectiveness of cognitive training beyond those children with ADHD, later studies trained adolescents who had extremely low birth weights (Løhaugen et al., 2011), adolescents with borderline intellectual disabilities (Van der Molen et al., 2010), and typically developing children with low working-memory capacities (Holmes et al., 2009). In all three studies, there was a significant effect of training. Especially promising, in one study training resulted in generalization to verbal learning tasks (Løhaugen et al., 2011), another resulted in generalization to arithmetic and story recall tasks (Van der Molen et al., 2010), and the other gains were maintained for 6 months post-training (Holmes et al., 2009), suggesting the possibility of long-term improvements in academic outcomes. More importantly, these studies demonstrate that cognitive training can improve the performance not only of children with diagnosed cognitive impairments such as ADHD or intellectual disabilities but also that of children without such a diagnosis but whose cognitive performance is lower than desired.

### Training attention

When looking to improve children’s attention, researchers typically focus on the particular skills of anticipation, ignoring distractors, conflict resolution, and/or response inhibition. When Rueda et al. (2005) trained all skills in 4- and 6-year-olds, they found significant changes in IQ scores for the 4-year-olds only. Examining the ERP data indicated that training effectively mimicked development for the 4-year-olds, but not the 6-year-olds (Rueda et al., 2004). Two studies that focused on training inhibitory control in 4-year-olds found conflicting results. Thorell et al. (2009) found no effect of inhibitory control training, though working-memory training was found to generalize to attention tasks. Conversely, Dowsett and Livesey (2000) found a significant benefit to inhibitory control training. The primary difference between the two studies is that Thorell et al. trained a random subset of preschool children whereas Dowsett and Livesey trained children who had previously been identified to struggle with inhibitory control, suggesting training benefits may be limited to those children who have attentional difficulties. Combined with the results of Rueda et al. (2004) to the extent that typically developing children do receive a benefit from attentional training, it may be limited to accelerating the developmental trajectory, with all children eventually attaining the same level. More research is needed to better understand the benefits of attentional training – both alone and in conjunction with working-memory training – but this preliminary evidence offers hope that attentional training would be beneficial for CI recipient children, who have already been shown to have a deficit relative to NH children.

The above studies offer hopeful cues for improving language outcomes in CI recipients while at the same time there remain questions that will need to be addressed. Beginning with the positive, it seems clear that cognitive training can improve cognitive functioning in children, particularly those children with a cognitive deficit. Having already demonstrated that CI recipient children have a cognitive deficit, it seems likely that cognitive training would be beneficial for this population. To the extent that cognitive skills support language functioning, it seems likely that improving cognitive functioning would lead to improved language functioning. Moving to the open questions, the type of training that has been used to assess the effectiveness of cognitive training to-date may not be the type of training that will result in optimal cognitive – and thereby language – improvement in CI recipient children. In particular, the effectiveness of cognitive training has generally been assessed using visuospatial training, but CI recipient children often score within the normal range on visuospatial executive function tasks and show greater deficits in the auditory domain (Dawson et al., 2002; Geers et al., 2009). There is the possibility that visuospatial cognitive training could transfer to address a cognitive deficit that exists primarily in the auditory domain (Blum and Yonelinas, 2001; Bherer et al., 2008), but the data are inconclusive (Vu et al., 2003; Schneider et al., 2011). There is also the question of whether unimodal training – which is the type that has typically been assessed – is optimal for addressing language deficits, which exist in a multimodal domain (Fagan and Pisoni, 2009; Bergeson et al., 2010). Together, then, though there is likely to be a benefit of cognitive training to improve cognitive performance in CI recipient children, there is the possibility that the type of training will need to be modified to address the fact that these deficits exist in the auditory domain.

## Training Cochlear Implant Recipients

Over the course of this manuscript we have made the case that CI recipient children’s delays in language learning are a result of the fact that they show deficits in auditory and cognitive processing relative to their NH peers and that these same skills in which the CI recipient children show a deficit are those skills on which language learning depends. We have also shown that both auditory and cognitive functioning can be improved via training, suggesting that it may be possible to improve language performance by improving the underlying skills in CI recipient children. The training data favors an approach that accounts for individual abilities in the training paradigm, and we believe such an approach will be most effective going forward. Along those lines, we recognize that some children may need more auditory training whereas other children may lag further behind in cognitive skills and require more training in this domain, and we suggest the development of training paradigms that focus on the needs of the particular child.

As a preliminary step into training CI recipient children, there has been a recent effort into improving the language outcomes via cognitive training (Kronenberger et al., 2011). Nine trainees were implanted by age three, were aged between 7 and 15 years at test, and showed average or lower working-memory performance on the BRIEF (Gioia et al., 2000) or the Children’s Memory Scale (Cohen, 1997). Children completed 5 weeks of Cogmed training following two baseline assessments. Following training, all working-memory measures except digit span backward improved relative to baseline assessments. No working-memory gains remained significant 6 months after training. The participants’ sentence repetition abilities improved significantly from baseline to post-test and these gains were maintained to the 6-month assessment.

Though this study provides preliminary support for cognitive training in CI recipient children, there remains room for improvement. There was improvement on the working-memory measures, but these gains were not maintained, possibly due to the fact that children scored in the average range on most measures prior to training (Kronenberger et al., 2011). In those cases where training gains have been maintained, children have started with lower than typical cognitive performance (Klingberg et al., 2005; Holmes et al., 2009; Van der Molen et al., 2010; Løhaugen et al., 2011). The language measure, sentence repetition, is proximally related to the training task, making it unclear the extent to which other language skills, such as story comprehension and phonological awareness, were improved. To the extent that successful language use depends on a battery of skills, post-training assessments will need to reflect the variety of abilities necessary and not just those abilities that are proximally related to the training program. We note again that it is likely that some children will require more auditory training than cognitive training and vice versa, and the type of training they receive should reflect their initial needs to result in optimal language learning outcomes. To this end, the fact that Cogmed is primarily a visuospatial training program was another limitation of the study, as it may not have addressed the auditory needs of the trainees.

## Conclusion

It seems unnecessarily simplistic to conclude this manuscript with the statement, “More work is needed,” and yet it is the case that more work is needed. We have shown that auditory and cognitive skills support language learning, suggesting that CI recipient children’s auditory and cognitive deficits lead to delays in language learning, but these claims remain to be tested explicitly. Work is needed to test the effectiveness of auditory training in the pediatric CI recipient population and more work is needed to better understand the effectiveness of cognitive training in CI recipient children. Work is also needed to determine the training program that will result in maximal long-term gains, possibly including occasional training tune-ups. As cognitive training efforts in CI children move forward, we advocate for an approach that emphasizes primarily auditory training – in contrast with Cogmed, which is primarily visuospatial – to better address the particular deficits of the trainees.

In this review we have repeatedly co-presented auditory and cognitive skills as supporting language function and as skills that can be improved via training. Yet it should not be inferred that we believe it necessary to train these two skills conjointly. If it is the case that auditory input is important for the development of auditory cognitive skills, then it is likely that the degree of benefit derived from cognitive training will be related to the trainee’s initial auditory processing ability. To that end, those CI recipient children who have relatively higher auditory skills – as a function of early implantation, early intervention, native ability, or some other factor – may show a greater benefit from cognitive training and thereby greater improvement on language measures than their peers with relatively poorer auditory ability. As a result, those children with relatively poorer auditory abilities are likely to require auditory training to improve their auditory skills prior to the onset of cognitive training but that children with greater auditory skills may be able to progress directly to cognitive training. Within the speech learning literature, there is an increasing awareness that matching the training paradigm to the learners’ pre-training individual abilities is likely to result in optimal training outcomes. Taking the lessons from the speech learning literature and applying them here would suggest that assessing children’s auditory and cognitive abilities prior to the administration of training could lead to more optimal training outcomes by determining the type of training needed, possibly eliminating auditory training for those children with relatively higher auditory abilities. Our proposal here for cognitive training to improve cognitive function and ultimately language outcomes in CI recipient children requires a better understanding of the causality between auditory and cognitive skills as well as a reliable predictor of relative auditory ability, returning us to the point that more work is needed.

Though the ultimate goal of any intervention is to improve language outcomes for CI recipient children, we should note that we do not think that training materials must necessarily be limited to speech or speech-like stimuli. Indeed, much promising work has shown a positive link between musical training and speech perception ability (Wong and Perrachione, 2007; Parbery-Clark et al., 2009; Besson et al., 2011; Perrachione et al., 2011). The auditory skills that musical training provides – including rhythm, timing, and sequencing – may provide a good foundation for the development of both cognitive and language skills. Additionally, though the focus of this review has been on spoken language perception, it should be noted that spoken language perception is a multimodal exercise, frequently requiring the integration of both auditory and visual input. Training CI recipient children to link the sounds they hear to the objects they perceive could improve both their language performance and their ability to successfully navigate their multisensory world.

Despite the fact that much work remains to be done, we are heartened by the progress that has been made training auditory and cognitive skills and the initial efforts to bring these training efforts to the CI population. We are optimistic that the additional efforts will result in a greater understanding of the contributions of auditory and cognitive skills to language learning and thereby to better training and rehabilitative paradigms.

## Conflict of Interest Statement

The authors declare that the research was conducted in the absence of any commercial or financial relationships that could be construed as a potential conflict of interest.
